# Identification of Urokinase Plasminogen Activator Receptor (uPAR)-Binding Peptides Using Phage Display: Strategy and Downstream Validation

**DOI:** 10.3390/cancers18203380

**Published:** 2026-10-19

**Authors:** Ane Beth Sloth, Ida Melisa Dawoodi, Tuule Treiberg, Anders Wilgaard Sinkjaer, Ida Vang Andersen, Amanda Oester Andersen, Emilie Caroline Skuladottir Bøgestad, Maria Rossing, Camilla Stavnsbjerg, Babak Bakhshinejad, Andreas Kjaer

**Affiliations:** 1 Department of Clinical Physiology and Nuclear Medicine & Cluster for Molecular Imaging, Copenhagen University Hospital—Rigshospitalet & Department of Biomedical Sciences, University of Copenhagen, 2200 Copenhagen, Denmark; 2 Department of Chemistry, University of Copenhagen, Thorvaldsensvej 40, 1871 Frederiksberg, Denmark; 3 Department of Otorhinolaryngology, Head & Neck Surgery and Audiology, Copenhagen University Hospital-Rigshospitalet, Blegdamsvej 9, 2100 Copenhagen, Denmark; 4 MDxCORE, Department of Clinical Biochemistry, Rigshospitalet, Copenhagen University Hospital, 2100 Copenhagen, Denmark; 5 Department of Clinical Medicine, Faculty of Health and Medical Sciences, University of Copenhagen, 2200 Copenhagen, Denmark

**Keywords:** biopanning, cancer, next-generation sequencing, peptide, phage display, urokinase plasminogen activator receptor, molecular imaging

## Abstract

**Background**: Peptides that selectively bind to cancer-associated proteins are attractive candidates for molecular imaging and targeted therapeutic applications. The urokinase plasminogen activator receptor (uPAR) represents a promising molecular target as it is highly expressed in several cancers and has been linked to cancer aggressiveness, invasion, and metastasis. This study applied an NGS-informed biopanning strategy against uPAR, combined with multi-assay downstream validation, to identify and prioritize candidate peptides. **Methods**: An optimized biopanning protocol incorporating additional selection controls was combined with next-generation sequencing to identify candidate uPAR-binding peptides from a phage display library. Selected peptides were evaluated using complementary in vitro assays, including flow cytometry and a quantitative plate assay. **Results**: Three peptide candidates were identified and selected for further evaluation with complementary in vitro assays. The optimized workflow incorporates measures to reduce, but not eliminate, enrichment of target-unrelated peptides, and includes complementary validation of the peptides in in vitro assays. Differences observed between the validation methods highlighted the importance of evaluating peptide candidates using complementary assays. **Conclusions**: This study demonstrates the value of combining an optimized phage display selection with next-generation sequencing and complementary validation assays for identifying candidate peptides. The identified peptides represent promising candidates for further optimization toward molecular imaging and targeted therapeutic applications.

## Simple Summary

Peptides binding specifically to receptors on the surface of cancer cells can be used for molecular imaging and targeted treatments. However, identifying peptides that bind to the intended target remains challenging because commonly used methods also select false-positive candidates. In this study, we sought to identify peptides with potential binding to the urokinase plasminogen activator receptor, a protein found at high levels in several cancers, and developed an improved biopanning strategy to reduce, although not eliminate, the risk of identifying target-unrelated peptides. Our approach combines peptide phage display with next-generation sequencing, additional selection controls, and several laboratory validation methods to improve the identification of genuine binding peptides. Using this strategy, we selected three candidate peptides for further evaluation of their behavior toward the urokinase plasminogen activator receptor. Our work provides a practical framework for discovering and validating new targeting peptides and may support the future development of more specific imaging agents and targeted therapies.

## 1. Introduction

Cancer incidence is on the rise with more than 19 million new cases and 10 million deaths globally in 2020 [1]. Consequently, better diagnostics and novel therapeutic interventions are needed. Conventional cancer treatments (surgery, radiotherapy, systemic chemotherapy) can be non-specific and cause damage to healthy tissue. Thus, more targeted treatments are needed. Cancer diagnosis relies on multiple tests, including biopsies and molecular imaging. For molecular imaging, the current gold standard is [^18^F]-Fluorodeoxyglucose ([^18^F]-FDG); however, this compound has also been shown to accumulate in inflammatory tissues and is therefore not specific to cancer [2,3,4,5].

The hallmarks of cancer outline a set of traits that cancers tend to acquire. These traits present potential avenues for diagnostic and therapeutic intervention [6,7]. The ongoing search to identify relevant cancer-specific biomarkers remains a focal point in cancer research. A biomarker and therapeutic target is the urokinase plasminogen activator receptor (uPAR). In most healthy cells, uPAR expression is low [8], but research has shown the overexpression of uPAR in multiple cancer types, including breast cancer [9], hepatocellular carcinoma [10], prostate cancer [11], and head and neck cancer [12]. Urokinase plasminogen activator (uPA) binds to uPAR, which localizes uPA to the cell surface and thereby facilitates the conversion of plasminogen into its active form, plasmin [13]. This proteolytic cascade initiates the degradation of extracellular matrix (ECM) proteins at the cell surface [14]. In addition to this proteolytic activity, uPAR modulates cell signaling through interactions with integrins and other cell-surface receptors [15,16]. Together, these proteolytic and signaling functions contribute to the role of uPAR in regulating cell adhesion and migration [17,18] and have been associated with increased tissue invasion, metastasis, and proliferation [19], traits that are considered among the hallmarks of cancer [6,7,20]. Importantly, high expression of uPAR has been associated with a poor prognosis for colorectal cancer [21], oral squamous cell carcinoma [22], gastric cancer [23], and invasive ductal breast carcinoma [24], among others.

Utilizing peptides as targeting agents offers advantages over antibodies. Peptides have more cost-effective manufacturing [25], are easily modified [26], and have rapid clearance [27]. A well-characterized example of a uPAR-targeting peptide is AE105, a 9-mer peptide antagonist that was originally identified through phage display selection against uPAR and subsequently optimized through affinity maturation [28]. AE105 binds uPAR with high affinity and has been extensively evaluated for molecular imaging [29,30,31] and therapy [32,33]. While AE105 remains a well-validated uPAR-targeting ligand, identifying additional candidate peptides may lead to peptides with different pharmacokinetic properties, which could offer advantages for both molecular imaging and therapy. The widely used technique of phage display has been instrumental in uncovering binders to desired targets [34,35]. Serving as a high-throughput approach, phage display relies on the use of diverse libraries containing phages with exogenous fusions of peptides linked to a coat protein [36]. In a process called biopanning, the phage display library is presented to the target of interest in iterative affinity selection rounds, which involve washing away unbound phages and recovery of bound phages. To decipher the peptide sequences of the isolated binders, the phage pool undergoes sequencing. Sanger sequencing of the eluted phage pool is still the most widely used method; however, more recently, next-generation sequencing (NGS) has been coupled with phage display, allowing the sequencing of thousands to millions of peptide sequences in parallel [37,38,39]. The use of NGS increases the sequencing depth and provides a higher throughput compared to Sanger sequencing.

While phage display offers potential for identifying novel peptide binders, it does carry inherent challenges. The main limitation of phage display selections is the emergence of target-unrelated peptides (TUPs). These TUPs fall into two categories: selection-related TUPs (Sr-TUPs), arising from binding to the components of the selection system (e.g., polystyrene), and propagation-related TUPs (Pr-TUPs), characterized by increased propagation capacity of phage clones, leading to their target-independent enrichment during the amplification rounds following each biopanning cycle [40,41]. It is important to identify and remove TUPs to increase the likelihood of successfully identifying a binding peptide in a phage display selection.

In this study, we exploited phage display to identify peptides exhibiting binding behavior to uPAR. While we have previously demonstrated at a methodological level how NGS can reveal propagation-driven artifacts during biopanning [42], the present work applies this insight to devise an NGS-informed biopanning strategy against uPAR with additional controls for the selection process, aiding the identification of candidate uPAR-binding peptides, followed by evaluation of candidate peptides using multiple in vitro assays. We report the discovery of peptides with favorable binding characteristics across multiple in vitro assays. Our findings demonstrate the value of integrating optimized selection strategies with NGS to improve peptide candidate selection, and underscore the importance of employing complementary in vitro assays, providing candidates for further optimization toward diagnostic and therapeutic applications.

## 2. Materials and Methods

### 2.1. Phage Display Peptide Library

Biopanning experiments were conducted using the Ph.D.^TM^-12 Phage Display Peptide Library (catalog #: E8111L, lot number: 10111202), purchased from New England Biolabs (NEB, Ipswich, MA, USA). The Ph.D.^TM^-12 library is based on the M13KE phage vector and expresses 12-mer peptides coupled to a GGGS-linker as N-terminal fusions to the pIII protein in a pentavalent manner. The library is reported to have a complexity on the order of 10^9^ independent clones [43].

### 2.2. Biopanning, Phage Titering, and Phage Amplification

Biopanning was performed against recombinant human uPAR protein (catalog #: PLAUR-3861H, Creative Biomart, Shirley, NY, USA) immobilized onto a MaxiSorp plate. The well was blocked with 0.5% BSA in 0.1 M NaHCO_3_ for 1 h and washed with TBST (TBS + 0.1% *v*/*v* Tween-20). The selection procedure was based on a subtractive approach. The biopanning included incubating the library in an empty well to remove potential plastic-binding peptides and incubating the supernatant from the previous step with 2% BSA. Lastly, the supernatant was incubated with the immobilized uPAR protein. The stringency was increased from the first to the second round by decreasing the amount of uPAR protein coated onto the well (from 5 μg to 2.5 μg), decreasing the incubation time of the phage library with the uPAR protein (from 1 h to 30 min), and increasing the subsequent number of washes (from 10 times to 20 times). A specific elution using 15 times the molar excess of recombinant uPA protein (catalog #: ENZ-264, Prospec Bio, Ness-Ziona, Israel) and a non-specific elution with 0.2 M Glycine-HCl, pH 2.2, and 1 mg/mL BSA were performed in parallel. The latter was neutralized by adding 15 μL of 1 M Tris-HCl, pH 9. A serial amplification of the unselected library was performed to identify and remove potential Pr-TUPs. The experiment was done by amplifying the unselected library in *Escherichia coli* (*E. coli*). Samples from each round were sequenced using NGS [44]. The identified peptides were used to filter out sequences from the biopanning samples that were potentially Pr-TUPs.

After each round of biopanning, the eluates were titered using plaque-count titration. Serial dilutions of the biopanning eluates were prepared in LB medium. The diluted samples were added to 200 μL of mid-log phase culture of the *E. coli* strain ER2738 (New England Biolabs). After 5 min of incubation, the infected bacterial cells were added to 3 mL of 45 °C top agar (0.7% LB agar) and plated onto pre-warmed LB agar plates containing Xgal (5-bromo-4-chloro-3-indolyl-β-D-galactopyranoside) and IPTG (isopropyl-β-D-1-thiogalactopyranoside) (Thermo Fisher Scientific, Waltham, MA, USA). Plates were inverted and incubated overnight at 37 °C. Blue plaques were counted the next day, and the titer in pfu/mL was determined.

Amplification of phage samples was performed to enrich the pool of binders to be used as input for the second round of biopanning. The biopanning eluates were added to a 1:100 diluted overnight culture of *E. coli* containing tetracycline, and the amplification was carried out for 5 h at 37 °C and 250 rpm. Precipitation was performed by centrifugation at 5000× *g* for 15 min at 4 °C. The supernatant, containing the bacteriophages, was added to 1/6 volume of PEG-NaCl buffer and allowed to precipitate overnight at 4 °C. The phages were pelleted by centrifugation at 12,000× *g* for 20 min at 4 °C and dissolved in 1× TBS.

### 2.3. Preparing Samples for Next-Generation Sequencing

The samples were prepared for sequencing as described previously [45,46]. The ssDNA from the phage pool was purified using the NucleoSpin ^®^ Plasmid Mini kit for plasmid DNA (Macherey-Nagel, Düren, Germany) following the manufacturer’s supplementary protocol for isolation of M13 DNA, without performing amplification in *E. coli*. The phage pellet was dissolved in RNase-free water (catalog #: 129112, Qiagen, Hilden, Germany). Per 1 mL of resuspended phage pellet, 10 µL of glacial acetic acid (catalog #: A6283, Sigma-Aldrich, St. Louis, MO, USA) was added, and the solution was mixed by inverting the tube. The sample was added to a NucleoSpin^®^ Plasmid Column placed in a Collection tube and spun for 1 min at 11,000× *g*. The NucleoSpin^®^ Plasmid Column was washed twice with Buffer AW before a final wash with Buffer A4. The DNA was eluted in RNase-free water and used as input for PCR. PCR was performed with up to 100 ng of ssDNA as input using the Q5 ^®^ High-Fidelity 2X Master Mix kit (New England Biolabs) and 0.5 µM of forward and reverse primers (Tag Copenhagen, Copenhagen, Denmark) in a 50 μL reaction volume. The primer sequences are listed below; the underlined nucleotides represent the target-binding region, and the remaining primer sequence is an overhang containing an adapter sequence.

Forward primer: 5’-TCG TCG GCA GCG TCA GAT GTG TAT AAG AGA CAG ACC TCG AAA GCA AGC TGA TAA ACC G-3’

Reverse primer: 5’-GTC TCG TGG GCT CGG AGA TGT GTA TAA GAG ACA GCT GTA GCA TTC CAC AGA CAG CCC-3’

The PCR program consisted of 98 °C for 30 s, followed by 5 or 10 cycles of 98 °C for 10 s, 60 °C for 30 s, 72 °C for 20 s, and a final extension for 2 min at 72 °C. The PCR products were purified by the GeneJET PCR Purification Kit (Thermo Fisher Scientific) according to the manufacturer’s protocol. As input for indexing PCR, 25 ng of PCR product was used, together with a unique combination of 5 μL each of the i5 and i7 Nextera XT indexing primers (Illumina, San Diego, CA, USA) using the Q5 ^®^ High-Fidelity 2X Master Mix kit. The PCR program consisted of initial denaturation at 95 °C for 3 min, 8 cycles of 98 °C for 30 s, 60 °C for 30 s, 72 °C for 30 s, and a final extension for 5 min at 72 °C. The indexed PCR products were purified using the GeneJET PCR Purification Kit. The formed products were analyzed using the BioAnalyzer 2100 DNA 1000 Kit (Agilent, Santa Clara, CA, USA) to verify that products of the correct sizes had been formed.

The samples were submitted to the Center for Genomic Medicine, Rigshospitalet, Copenhagen University Hospital, Denmark. There, the samples underwent quality control by the Fluoroskan^TM^ Microplate Fluorometer with the Quant-iT^TM^ 1X dsDNA HS Assay Kit (Thermo Fisher Scientific). The indexed samples were sequenced using a NextSeq 500/550 Mid Output Kit v2.5 (Illumina) by single-end sequencing of 250 bp reads. Adapters were trimmed, and the individual base call (BCL) files were demultiplexed to generate FASTQ files using the Illumina bcl2fastq software (v2.20). Quality control, sequencing, and generation of FASTQ files were conducted at the Center for Genomic Medicine.

### 2.4. Analysis of Next-Generation Sequencing Data

A Python script developed in-house was used to process the FASTQ files. The script contained several control steps, including the removal of reads with three nucleotides containing a Phred score below Q20, sequences not containing the GGGS-linker, and sequences containing ‘*’ or an ‘X’. The cleaned data were converted into individual .csv files. The relative peptide frequency of each peptide sequence was calculated as the number of reads assigned to a given sequence divided by the total number of reads in the given phage pool. The relative frequencies were used for calculating the enrichment factor (EF), defined as the ratio of the relative frequency of a peptide in the biopanning sample to the relative frequency of the peptide sequence in the unselected library. This allows comparison of the sequence representation across different samples and provides a more accurate measure of the sequence enrichment during the selection compared with absolute read counts, which are affected by the sequencing depth of the individual sample.

### 2.5. Selecting Peptides and Peptide Synthesis

Sequences exhibiting a high EF in the uPAR biopanning samples and being either absent from or present at a low frequency in the serial amplification control experiment were selected. Peptides 1–8 were not found in any serial amplification sample, whereas peptide 9 was found once in one serial amplification sample, peptide 10 was found three times in one serial amplification sample, and peptide 11 was found 6 times in one serial amplification sample and twice in another. The selected peptide sequences were subsequently investigated using prediction tools. The peptide solubility was estimated using PepCalc.com—Peptide Property Calculator from Innovagen [47]. The peptides were analyzed using the tools in the SAROTUP [48] and Biopanning Data Bank (BDB) databases [49], to determine whether any of the peptide sequences had previously been identified in other biopanning experiments.

The peptides were synthesized using solid-phase synthesis by Wuxi AppTec (Shanghai, China) in an NTA-salt form. The peptides were synthesized with a PEG2 linker and fluorescently labeled with fluorescein isothiocyanate (FITC) at the N-terminus. The peptides were dissolved in PBS with up to 10% (*v*/*v*) MeCN to aid solubility.

### 2.6. Determining the uPAR Expression in Positive and Negative Control Cell Lines

A qPCR assay was employed to determine the gene expression of *PLAUR* in the two cell lines, Jurkat (Clone E6-1, TIB-152^TM^, ATCC, Manassas, VA, USA) and SUP-T1 (CRL-1942^TM^, ATCC). RNA was extracted from cell pellets using a QIAcube (Qiagen) with the RNeasy Mini QIAcube Kit (Qiagen, catalog #: 74116). The cDNA synthesis was performed with 1000 ng of RNA as input using the High-Capacity RNA-to-cDNA^TM^ Kit (catalog #: 4387406, Thermo Fisher Scientific). The qPCR was run using the TaqMan Fast Advanced Master Mix for qPCR (catalog #: 4444556) together with TaqMan^®^ assays (containing forward and reverse primers as well as a TaqMan^®^ probe) for *PLAUR* (Assay ID: Hs00958880_m1) and *GAPDH* as the housekeeping gene (Hs02786624_g1). The normalized gene expression was calculated using the 2^−ΔΔCt^ method.

To verify uPAR expression, flow cytometry was performed by washing the cell pellets in FACS buffer (PBS with 0.5% BSA, 2 mM EDTA, and 0.1% NaN_3_). The cell pellet was resuspended in Human BD Fc Block™ (BD Biosciences, Franklin Lakes, NJ, USA) and incubated for 5 min on ice. Cells were then stained with a PE-conjugated anti-uPAR (CD87) monoclonal antibody (catalog #: 555768, clone VIM5, BD Biosciences) for 30 min on ice in the dark, together with eBioscience^TM^ Fixable Viability Dye eFluor^TM^ 780 (eBioscience, San Diego, CA, USA). The cells were washed in FACS buffer and run on the BD Fortessa X-20 flow cytometer (BD Biosciences). Data were analyzed using FlowJo (BD Biosciences, version 10.9.0).

### 2.7. Cell-Based Validation of Peptide Binding Capacity

#### 2.7.1. Flow Cytometry

To assess peptide binding, flow cytometry was performed by blocking the cells in 1% protease-free bovine serum albumin (BSA) (Merck KGaA, Darmstadt, Germany) in PBS for 1 h on ice. The cells were stained with 10 μM of each peptide together with eBioscience^TM^ Fixable Viability Dye eFluor^TM^ 780 (eBioscience) in the dark for 1 h on ice. The cells were washed and run on the BD Fortessa X-20 flow cytometer. Data were analyzed using FlowJo (BD Biosciences, version 10.9.0), and the normalized binding values were determined as the ratio of median fluorescence intensity (MFI) of the FITC+ signal for the peptide signal to the MFI of FITC+ signal for the samples treated only with FITC.

#### 2.7.2. Quantitative Fluorescent Plate Assay

To evaluate binding of the peptides using another assay based on fluorescence, a quantitative plate assay was conducted. Jurkat and SUP-T1 cells were harvested, and 100,000 cells/well were added to a 96-well U-bottom plate (Nunc (Thermo Fisher Scientific), Rochester, NY, USA). The cells were washed and blocked in 1% protease-free BSA for 1 h on ice. The cells were incubated with 10 μM peptide solutions for 1 h on ice in the dark. Lastly, the plate was washed and transferred to a black Nunc ^®^ MicroWell 96-well optical bottom plate (Sigma-Aldrich), and the fluorescence was measured using a plate reader (FLUOstar Omega Microplate Reader, BMG, Ortenberg, Germany) with gain adjustment for the well with the highest fluorescent signal.

#### 2.7.3. Statistical Analysis

Statistical analysis was performed using GraphPad Prism (version 11.0.0). For the flow cytometry and quantitative plate assay data, a two-way ANOVA was performed with peptide identity and cell line as factors, followed by Tukey’s multiple comparisons test for post hoc pairwise comparisons. Main effects of peptide and cell line, as well as the peptide × cell line interaction, are reported alongside the post hoc results. A significance threshold of α = 0.05 was used throughout.

### 2.8. Surface Plasmon Resonance

Surface plasmon resonance (SPR) was performed to investigate the peptides (6, 7, and 10) binding to the same recombinant protein used for biopanning. All SPR analyses were carried out on a Biacore^TM^ X100 instrument on the sensor chip CM5 (Cytiva, Marlborough, MA, USA). 1× PBS supplemented with 50 µM EDTA and 0.01% (*v*/*v*) surfactant P-20 was used as running buffer, and experiments were carried out at 25 °C. A concentration of 20 µg/mL of human recombinant uPAR protein (catalog#: PLAUR-3861H, Creative Biomart) in 10 mM sodium acetate, pH 5.0 was covalently immobilized on the sensor chip via random amine coupling using *N*-hydroxysuccinimide/1-ethyl-3-(3-dimethylaminopropyl) carbodiimide hydrochloride for surface activation and 1 M ethanolamine-HCl pH 8.5 for deactivation of the excess activated surface after ligand addition. Immobilization levels between 900 and 1100 RU were obtained. Human recombinant CD4 protein (catalog #:158-10400-H08H-100, Nordic Biosite, Täby, Sweden) was used as a reference protein. A concentration of 23 µg/mL of human recombinant CD4 protein in HBS supplemented with 0.01 M HEPES, 0.15 M NaCl, 0.03 M EDTA, and 0.05% (*v*/*v*) surfactant P-20 (HBS-EP+) was immobilized using an identical approach as for immobilization of uPAR (random amine coupling using HBS-EP+ as the running buffer). An immobilization level of approx. 600 RU was obtained.

A blank immobilization was performed on the reference surface by activating and deactivating the surface in the same manner as before, with no addition of ligand. The integrity of the uPAR-immobilized chip was tested with AE105 as the positive control. To determine peptide binding capability to uPAR and a reference protein (CD4), two blank injections of running buffer followed by an injection of 0.5 μM and 5 μM of each of the peptides for both the uPAR and the CD4 chip, were performed with an association time of 120 s and a dissociation phase of 120 s at a flow rate of 30 µL/min flow rate. Each injection was followed by an injection of 0.1 M acetic acid in 0.5 M NaCl for sensor chip regeneration. Analyses were performed in duplicate; however, due to precipitation of peptide 10, only one experimental run was performed on the uPAR chip. The Biacore^TM^ X100 Evaluation Software version 2.0.2 was used to analyze the binding results. All analyzed data were double referenced to correct for drifts, artifacts, and non-specific binding.

### 2.9. Blood Stability Assay

A blood stability assay was conducted by drawing a fresh blood sample from a healthy individual in 9 mL BD Vacutainer^®^ EDTA tubes (BD Biosciences). The assay protocol was designed based on previously published methods for assessing peptide stability in blood [50,51,52]. FITC-labeled peptides were added to yield a total concentration in the blood sample of 2.26 µM. The blood samples were incubated at 37 °C in the dark for the duration of the experiment. The sample serving as the background was collected before adding the peptide. Samples were collected after incubation in blood for 0 h, 1 h, 3 h, 4 h, 6 h, 8 h, 10 h, and 24 h. For each sampling, a volume of 750 µL was taken out and spun down for 5 min at 16,000× *g*. The plasma layer was mixed with 15% TCA (CAS Number: 76-03-9, Serva, Heidelberg, Germany) and incubated for 10 min on ice. The sample was centrifuged for 5 min at 16,000× *g*. The supernatant was adjusted to pH 7 and kept at −20 °C until analysis by HPLC.

A 300 µL sample was placed into an HPLC system consisting of a Gilson Aspec XL autosampler with a 5 mL Rheodyne injector loop, a Gilson 306 pump, and a Dionex P480 pump. After injection, the sample was passed through a 2 µm inline filter before entering a Zorbax SB-C18 HPLC column (5 µm, 4.6 × 150 mm, Agilent). The column was connected to a Dionex UltiMate^TM^ 3000, RS Fluorescence Detector. The HPLC system was controlled by the Chromeleon HPLC software version 7.2.9.11323 (Dionex Corporation, Sunnyvale, CA, USA). The flow rate was set to 1.5 mL/min, and a gradient was applied from 5% to 100% solvent B in solvent A (H_2_O/MeCN/TFA, A: 100/0/0.1%, B: 0/100/0.1) over 1–7 min and after 2 min returning to 95% A in 1.5 min with a total runtime of 15 min. Fluorescence detection was performed with excitation: 490 nm and emission: 520 nm, with a sensitivity setting of 7. The peptide percentage at each time point was determined by HPLC peak area normalization at the peptide-specific retention time, expressed as the percentage of the total integrated peak area of detected peaks in the chromatogram, i.e., the reported peptide percentage represents the relative proportion of the HPLC signal corresponding to the intact peptide at each time point.

### 2.10. Molecular Docking

Molecular docking was performed to predict the binding site of the identified peptide sequences. PDB files containing the predicted peptide conformations were obtained via PEP-FOLD 3.5 [53]. Polar hydrogens were added to all peptides via PyMOL (version 2.5.2) [54], and a PDBQT file was created using AutoDockTools (version 1.5.7) (ADT) [55]. The crystal structure of the AE147-bound uPAR (PDB code: 1YWH) [56] was used for this study. It was visualized and slightly modeled in PyMOL by isolating one uPAR-AE147 moiety, removing water molecules, removing the bound AE147, and adding polar hydrogens before creating a PDBQT file in ADT. The receptor was treated as rigid while the peptide ligands were treated as flexible, with all their rotatable bonds sampled during docking. The peptide-uPAR docking area was defined via a 1.0 Å spaced grid box totaling 162,400 Å^3^ (x = 58 Å, y = 50 Å and z = 56 Å) containing the active binding site of uPAR centered on the receptor (x = 70.9 Å, y = −28.4 Å, and z = 40.4 Å). The docking was performed using AutoDock Vina (version 1.1.2) [57], using the default Vina scoring function (exhaustiveness = 8, num_modes = 9, energy_range = 3 kcal/mol). The highest-ranked pose based on the affinity parameter (kcal/mol) for each peptide was selected for presentation and analysis, with the resulting docking scores of −6.9 kcal/mol for peptide 6, −6.8 kcal/mol for peptide 7, and −5.8 kcal/mol for peptide 10.

## 3. Results

### 3.1. Identification of Peptide Sequences

**Figure 1:**
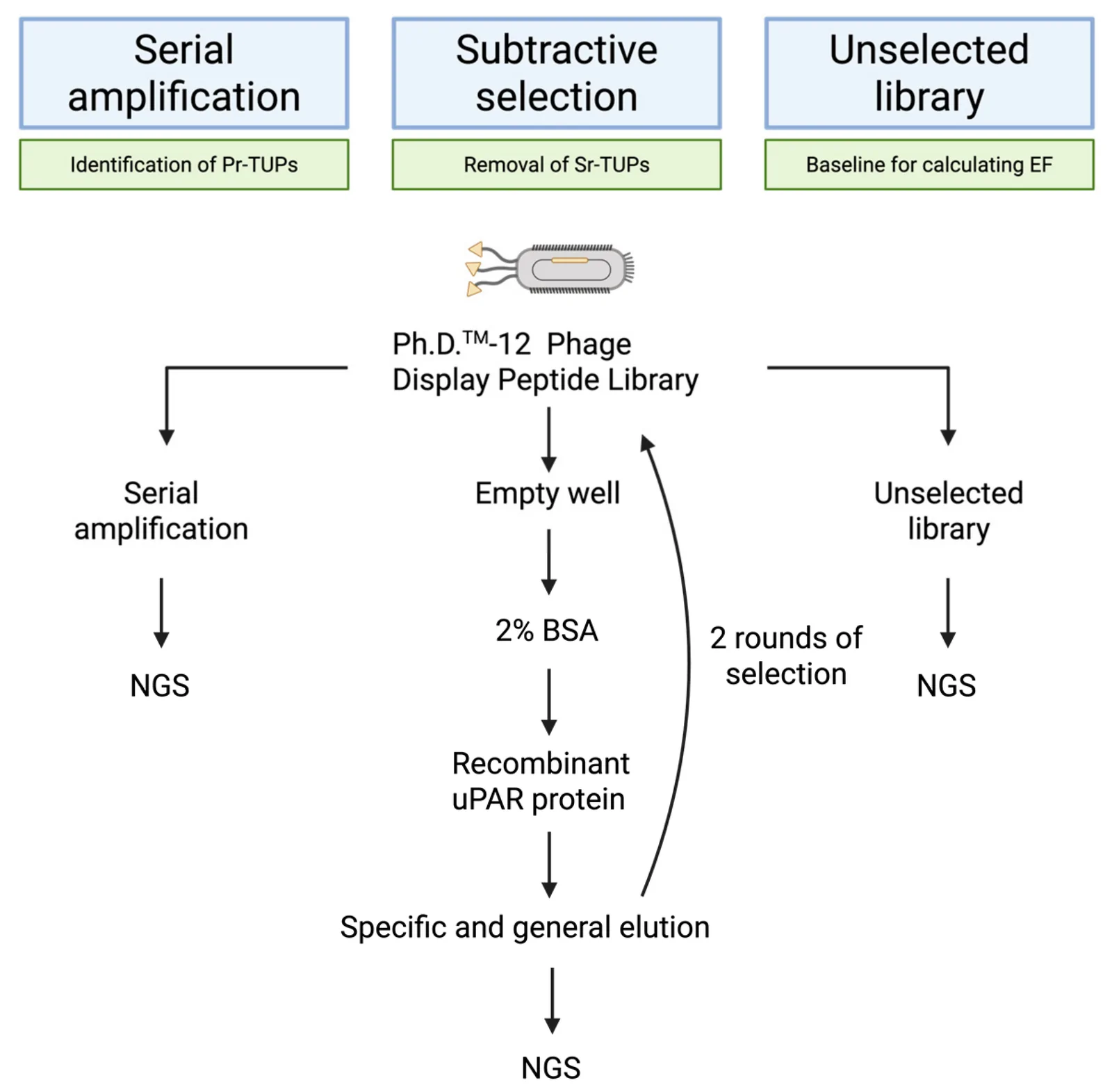
Overview of the biopanning strategy. The experiment consisted of a subtractive biopanning toward recombinant uPAR protein. The Ph.D.^TM^-12 Phage Display Peptide Library underwent a subtractive biopanning toward recombinant uPAR protein, with steps included to remove Sr-TUPs (selection-related target-unrelated peptides), and a serial amplification control experiment to identify and filter out Pr-TUPs (propagation-related TUPs). The unselected library was also included as a control and used as the baseline for calculating the enrichment factor (EF). Although two rounds of biopanning were performed, the peptides were selected from the output of the first round. NGS: next-generation sequencing, BSA: bovine serum albumin.

A biopanning experiment against recombinant uPAR protein was conducted using the Ph.D.^TM^-12 Phage Display Peptide Library. The experiment included subtractive biopanning, in which the phage display library was presented to an empty MaxiSorp plate, and subsequently to BSA to eliminate Sr-TUPs. A separate experiment with serial amplification of the naïve library was used as a control to remove suspected Pr-TUPs from the dataset. An overview of the biopanning experiment is provided in Figure 1.

The NGS datasets underwent quality filtering. The results of the quality filtering are shown in Table 1. For each sample, a high proportion of reads were removed. Some removed reads did not meet the requirements for the quality score, i.e., a read containing three nucleotides with a Phred score below Q20. Some reads were removed due to the presence of an ‘X’, ‘*’, or absence of the GGGS-linker. The number of reads removed due to different reasons is shown in Table 1. The cleaned data for each sample were merged into one file, which also included the serial amplification datasets. This enabled identification of peptides in the biopanning samples with no or low presence in the serial amplification experiment. Furthermore, the enrichment factor (EF), defined as the ratio of the relative peptide frequency in the biopanning sample to its relative frequency in the unselected library, was calculated for all peptides and used as a criterion for their selection. Peptides identified using different types of elution (acidic or uPA) were selected. Peptides with a high EF as well as a low or no presence in the serial amplification datasets were favored. Peptide selection was based exclusively on round 1 biopanning data, as round 2 eluates showed a marked reduction in sequence diversity, dominated by a single clone (different from another selected peptide by a single amino acid) together with a high proportion of M13KE phage without insert, resulting in low EF values for the previously identified candidates. The complete list can be seen in Table 2.

Furthermore, in silico predictions of the peptides were performed. Analysis using the tools in the SAROTUP and BDB databases revealed that none of the peptides had been identified in a previous biopanning experiment, although some peptides contained motifs that had been identified for other targets. Additionally, it was found that some peptides were predicted to have poor solubility. However, these were all included for in vitro testing. The results are summarized in Table A1.

### 3.2. Selection of Suitable Cell Lines

**Figure 2:**
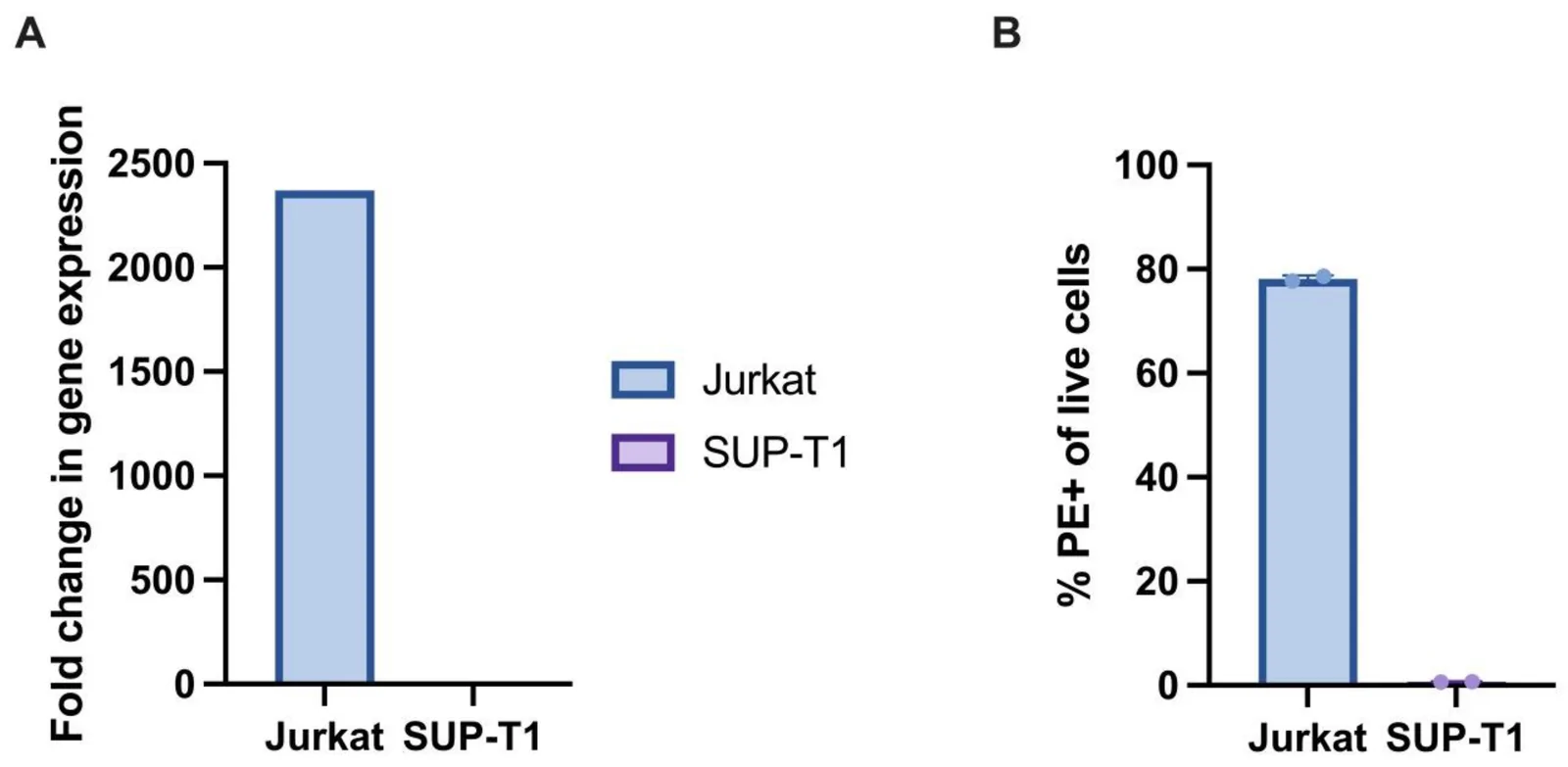
Selecting Jurkat as the uPAR-positive cell line and SUP-T1 as the cell line with lower uPAR expression for cell-based assays. (**A**) qPCR assay of Jurkat (blue) and SUP-T1 (purple) showing the fold change in gene expression, using the 2^−ΔΔCt^ method, normalizing to *GAPDH*. (**B**) Flow cytometry of cell lines, Jurkat (blue) and SUP-T1 (purple), stained with a PE-conjugated uPAR antibody. The percentage of PE-positive cells is shown. Data are shown as mean ± SD, and n = 2.

**Figure A1:**
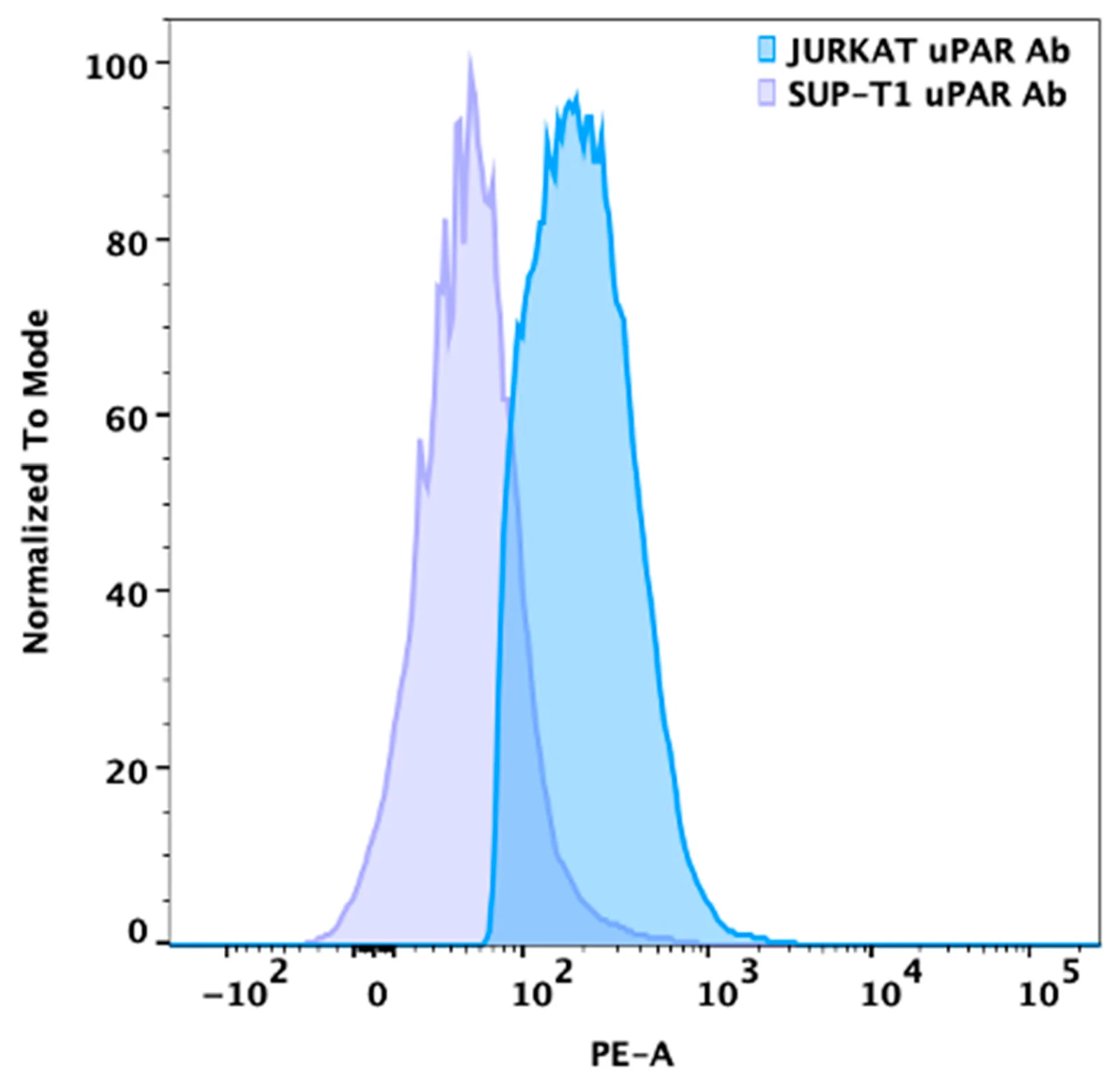
Histograms of median fluorescence intensity from one flow cytometry experiment with antibody staining of Jurkat (blue) and SUP-T1 (purple) normalized to mode.

A qPCR assay was performed to assess the difference in *PLAUR* gene expression between the two cell lines, Jurkat and SUP-T1. The results in Figure 2A show that Jurkat cells have more than a 2300-fold higher gene expression of *PLAUR* than SUP-T1. Flow cytometry was conducted to assess the difference in protein expression between Jurkat and SUP-T1 cells. The results in Figure 2B show a clear difference in PE+ cells for Jurkat cells compared to SUP-T1 cells. The results for one experiment, shown as histograms can be seen in Figure A1.

### 3.3. Cell-Based Assays

Flow cytometry was used to assess binding of the dissolved FITC-labeled peptides to the two cell lines, Jurkat and SUP-T1. Peptides 1 and 8 could not be brought into solution and were therefore excluded from the binding validation experiments. This poor solubility was consistent with the peptide solubility predictions (Table A1).

**Figure 3:**
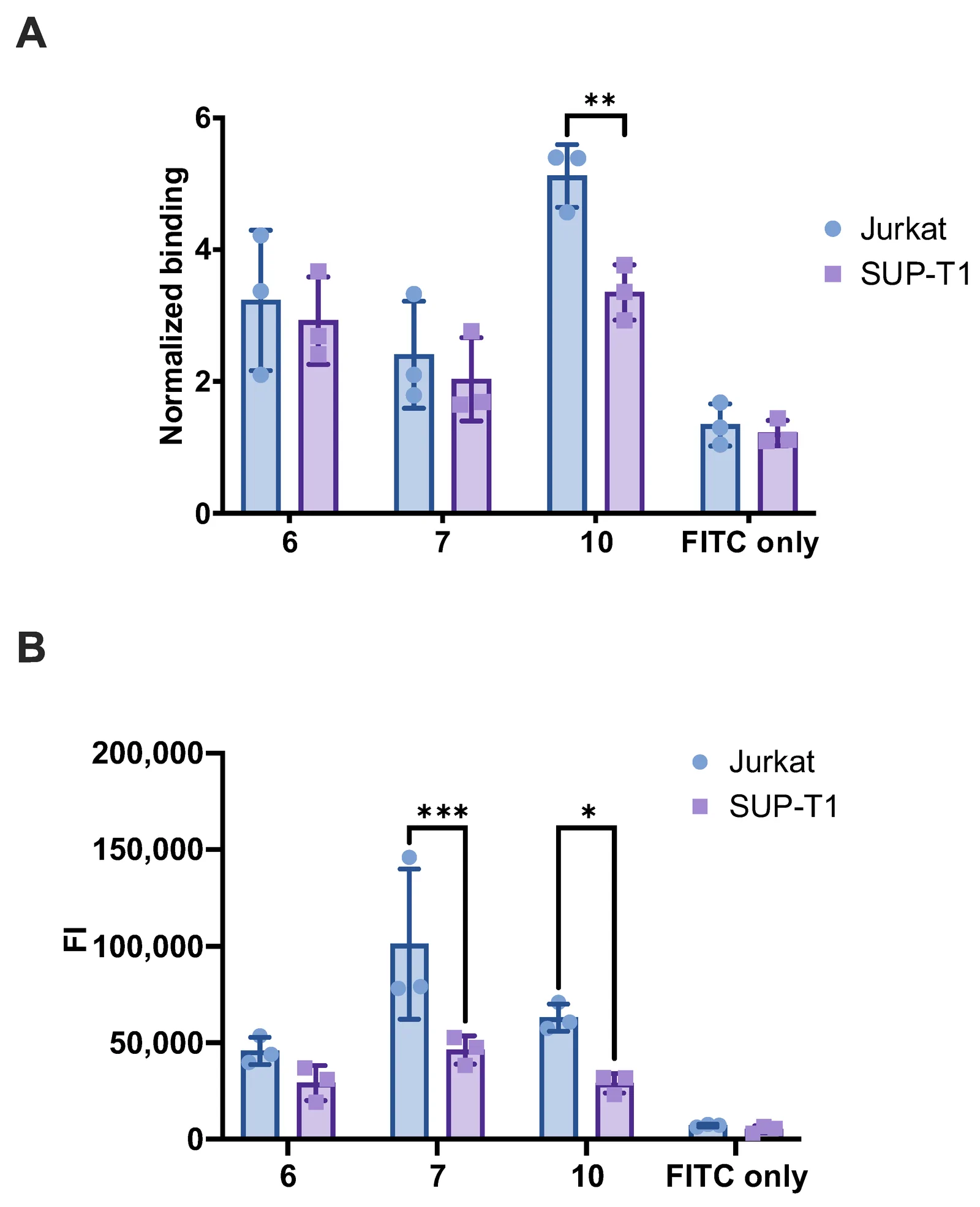
Validation of peptide binding behavior using flow cytometry and a quantitative plate assay. (**A**) Data from flow cytometry analysis of peptides 6, 7, and 10, including pure FITC as a negative control. The two cell lines Jurkat (blue) and SUP-T1 (purple) were included as cell lines with high and lower uPAR expression, respectively. The normalized binding for the two cell lines was compared using a two-way ANOVA and post hoc Tukey’s test. ** represents *p* < 0.01. The data are shown as mean ± SEM, and n = 3 biological replicates. (**B**) Data from the quantitative plate assay of peptides 6, 7, and 10, including pure FITC as a negative control. The two cell lines Jurkat (blue) and SUP-T1 (purple) were included as cell lines with high and low uPAR expression, respectively. The fluorescence intensity (FI) signal was compared using a two-way ANOVA and post hoc Tukey’s test. * represents *p* < 0.05, *** represents *p* < 0.001. Data are shown as mean ± SEM, and n = 3 biological replicates.

**Figure A2:**
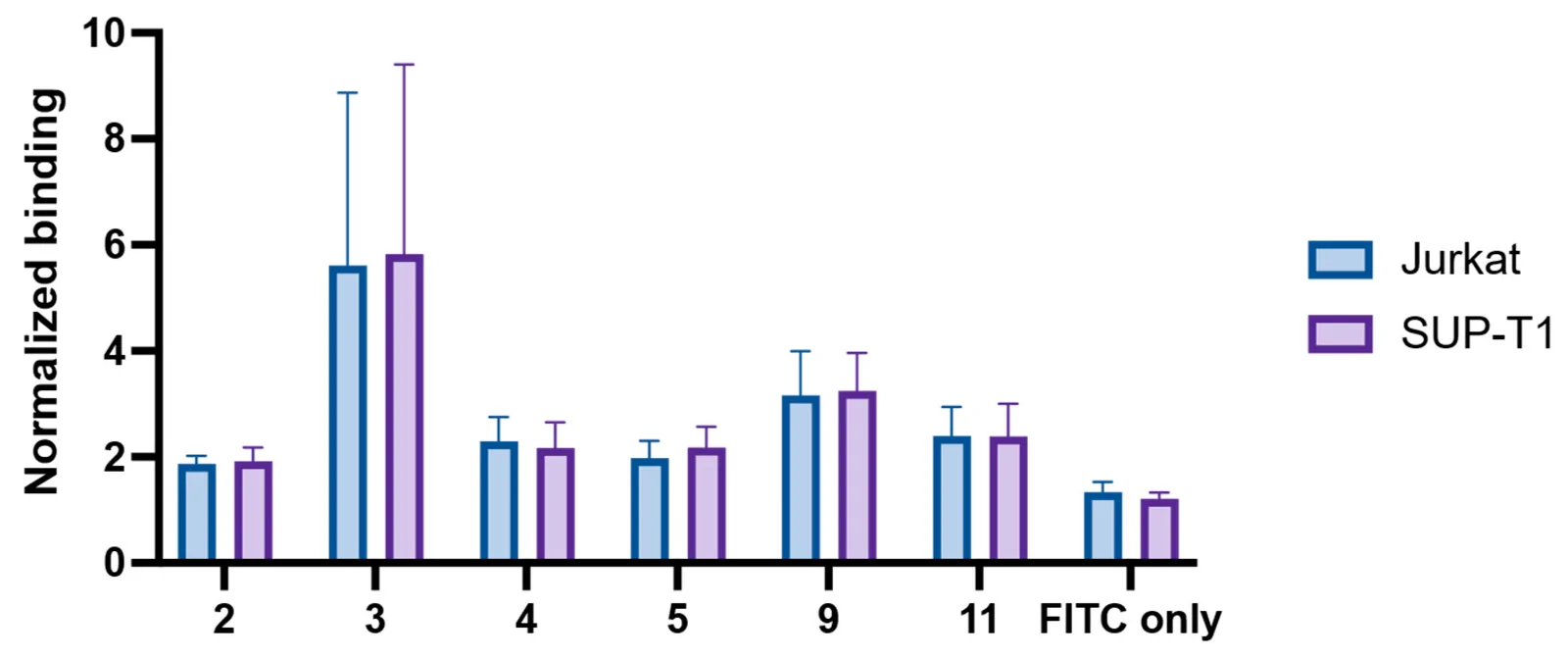
Data from flow cytometry experiments performed using all peptides that could be dissolved. The normalized binding of each peptide is shown on the y-axis and was calculated as the ratio of the median fluorescence intensity (MFI) of the FITC signal for each peptide to the MFI of the FITC-only control. This experiment was conducted using Jurkat cells, a cell line with higher uPAR expression, and SUP-T1, a cell line with lower uPAR expression. For each peptide, 10 µM was added. As a negative control, 10 μM pure FITC was included. This experiment was performed to select the peptide candidates for subsequent testing. The peptides shown in this plot were the ones that were not selected for subsequent analysis. Data are shown as mean ± SEM, and n = 2.

**Figure A3:**
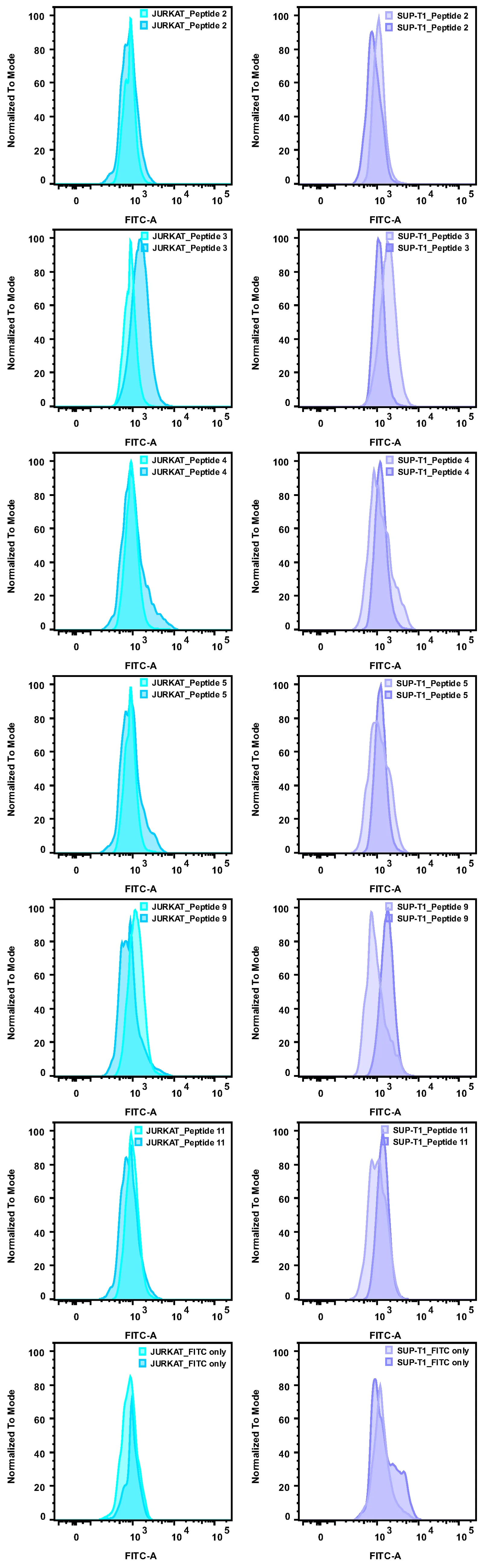
Data from flow cytometry experiments performed using all peptides that could be dissolved. The histograms from each experiment (n = 2 biological replicates) for each peptide are shown for a cell line with high uPAR expression, Jurkat cells (blue), and for a cell line with low uPAR expression, SUP-T1 (purple). The histograms are shown normalized to mode. For each peptide, 10 µM was added. As a negative control, 10 μM pure FITC was included. This experiment was performed to select the peptide candidates for subsequent testing. The peptides shown in this plot were the ones that were not selected for subsequent analysis.

**Figure A4:**
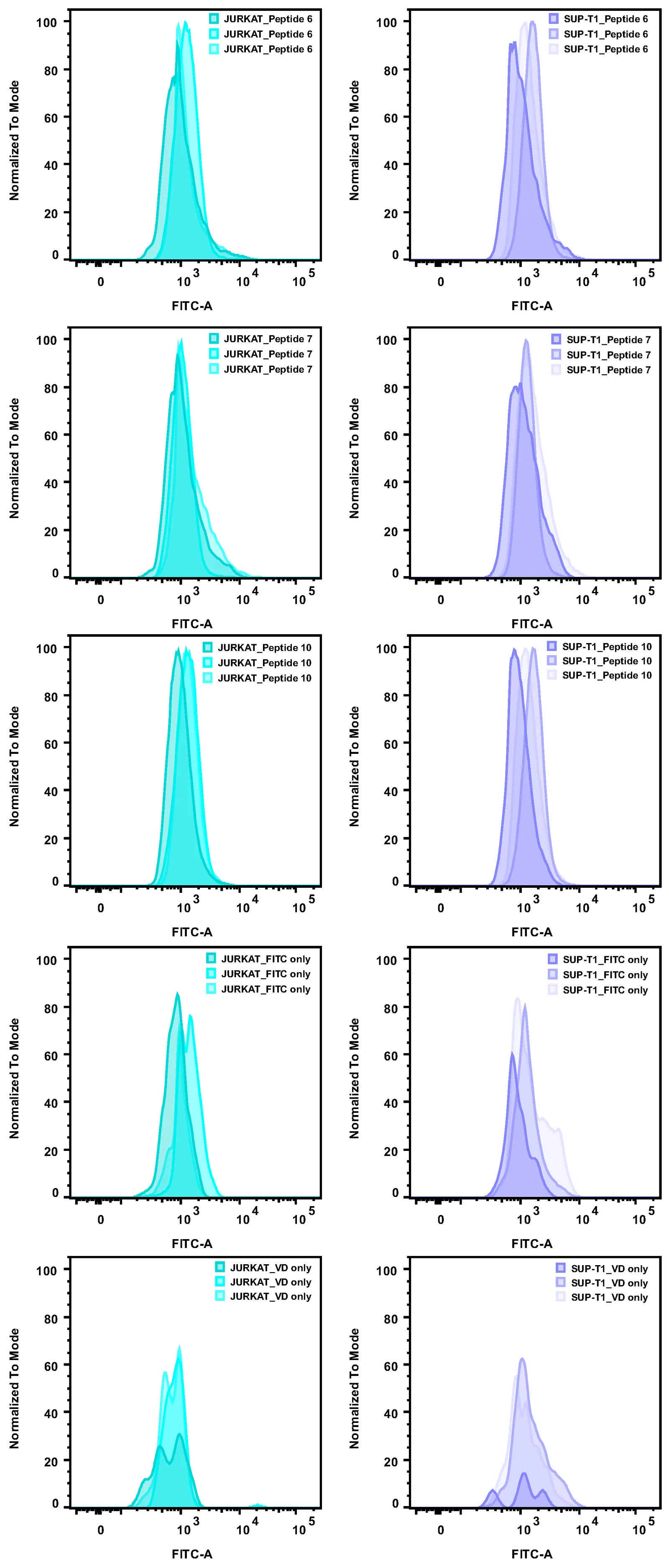
Data from flow cytometry experiments using the three selected peptides. The histograms from each experiment (n = 3 biological replicates) for each peptide are shown for a cell line with high uPAR expression, Jurkat (blue), and for a cell line with low uPAR expression, SUP-T1 (purple). The histograms are shown as normalized to mode. For each peptide, a concentration of 10 µM was added. As a negative control, 10 µM pure FITC was included. A sample with only viability dye was included as well.

**Figure A5:**
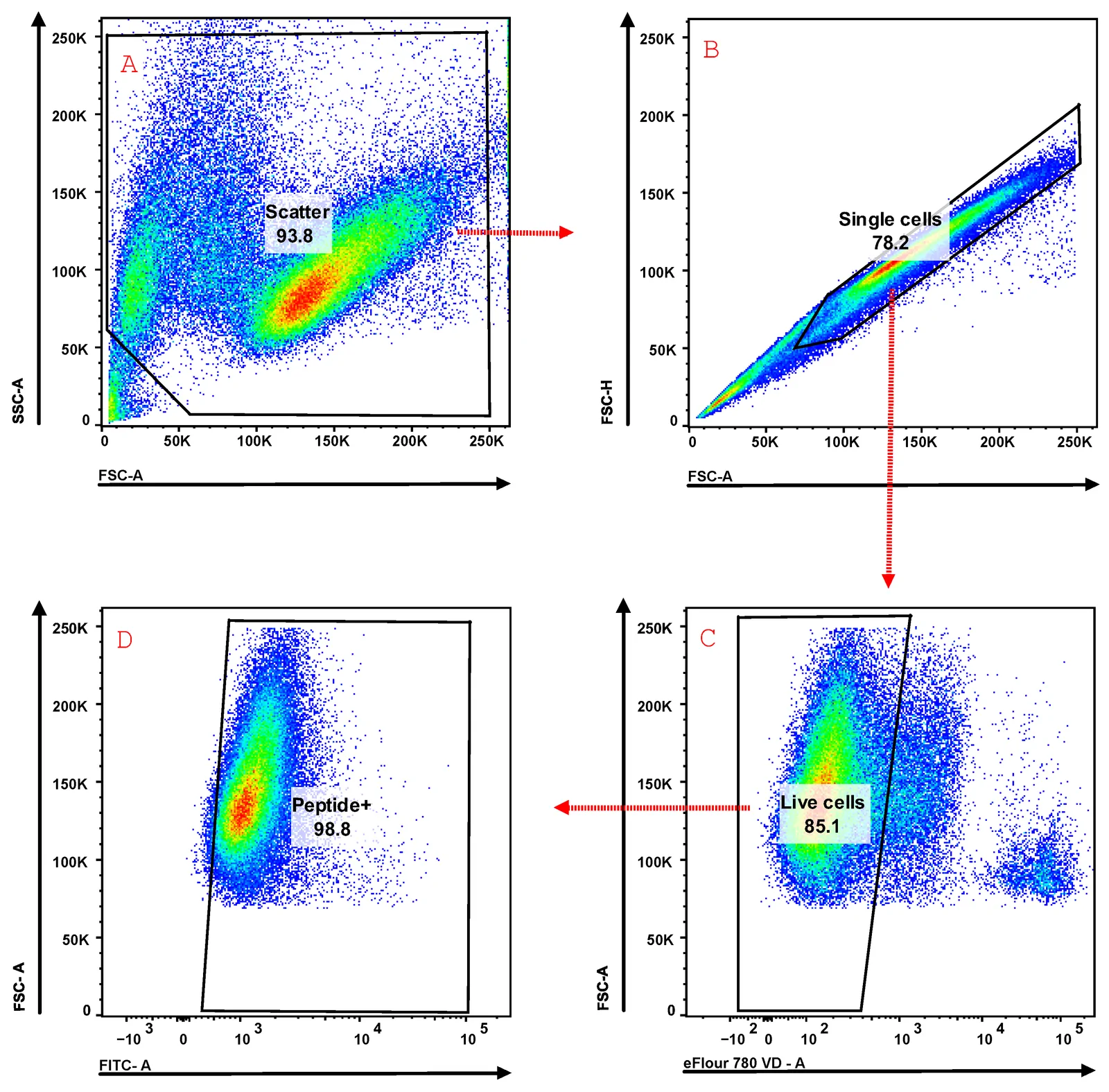
An overview of the gating strategy employed to analyze flow cytometry data of peptide binding. (**A**) The plot shows the scatter data. Forward scatter-area (FSC-A) on the x-axis and side scatter-area (SSC-A) showed all acquired events. Gates were set to exclude cell debris. (**B**) The plot shows the data when plotting FSC-A and forward scatter height (FSC-H). The gates were set to exclude duplicate cells or cell clusters and only select single cells. (**C**) The plot shows the data when plotting FSC-A and eFluor viability dye (VD). Gates were set to exclude dead cells and only include live cells in the subsequent analysis. (**D**) The plot shows the data when plotting FSC-A and FITC-A, as the peptides were conjugated to FITC.

Initial tests with all dissolvable peptides were performed using flow cytometry. Of the nine peptides, six showed no difference between the two cell lines, as shown in Figure A2 and Figure A3. These peptides were not selected for subsequent analyses. Peptides showing an increased FITC signal in Jurkat cells compared to SUP-T1 cells were included in replicate experiments. All replicates (n = 3) are combined in Figure 3A. The data are shown as histograms in Figure A4. A two-way ANOVA of the flow cytometry data (peptides 6, 7, 10, and a FITC-only control) revealed a significant main effect of the peptide (F(3,16) = 23.90, *p* < 0.0001) and a significant main effect of the cell line (F(1,16) = 6.240, *p* = 0.0238); however, the peptide × cell line interaction was not statistically significant (F(3,16) = 2.165, *p* = 0.1321). Post hoc Tukey’s comparisons indicated a statistically significant difference between Jurkat and SUP-T1 specifically for peptide 10 (*p* = 0.0034). Peptides 6 and 7 also showed increased normalized binding in Jurkat cells compared to SUP-T1, but the difference was not statistically significant. The gating strategy for the data is shown in Figure A5.

A quantitative plate assay was performed to assess the binding of the three peptides (6, 7, and 10) using another fluorescence-based setup. The results in Figure 3B show that both peptides 7 and 10 exhibit a significant difference in fluorescence intensity between Jurkat and SUP-T1 cells. A two-way ANOVA of the plate assay revealed a significant main effect of the peptide (F(3,16) = 20.93, *p* < 0.0001), a significant main effect of the cell line (F(1,16) = 19.55, *p* = 0.0004), and a significant peptide × cell line interaction (F(3,16) = 3.511, *p* = 0.0397), indicating that the magnitude of the difference in signal between Jurkat and SUP-T1 varied significantly across peptides.

**Figure A6:**
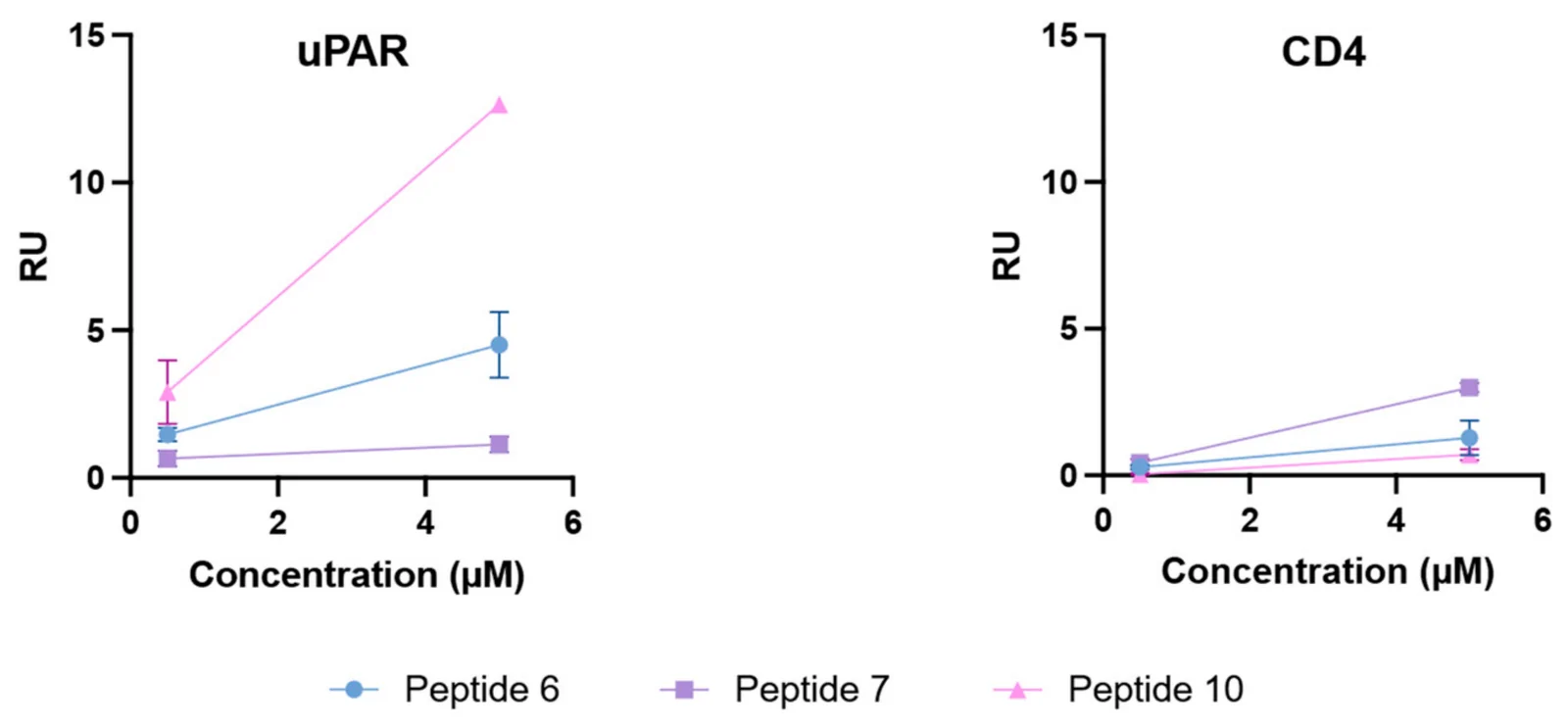
Results from surface plasmon resonance (SPR) experiments. The graph illustrates the highest signal in response units (RU) obtained from the SPR analyses. The FITC-labeled peptides 6, 7, and 10 were investigated. A high and a low concentration were tested for each peptide. A reference chip containing an unrelated protein (recombinant human CD4) was included as a control. All experiments were performed in duplicate (except for analyses with peptide 10 on the uPAR chip due to poor solubility).

**Figure A7:**
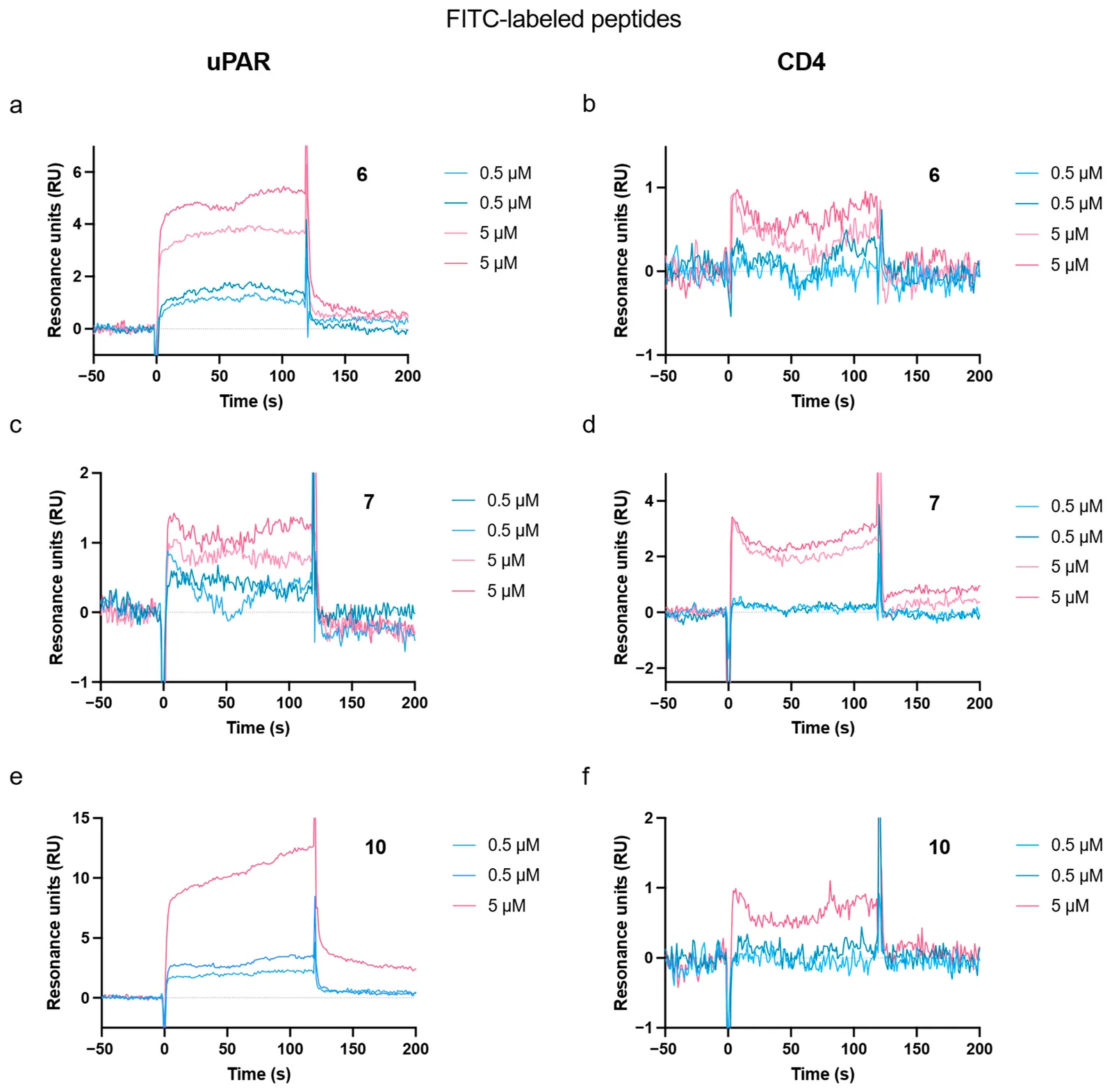
Sensorgrams from surface plasmon resonance (SPR) analyses of FITC-labeled peptides run on a uPAR chip (**a**,**c**,**e**) and a reference protein (CD4) chip (**b**,**d**,**f**). Concentrations of the peptides were 0.5 μM (blue) and 5 μM (red), run in duplicate (only one replicate for peptide 10 as it fell out of solution).

The binding of the FITC-labeled peptides to the same recombinant protein used for biopanning was investigated using surface plasmon resonance (SPR). Each peptide was tested at 0.5 µM and 5 µM. The results are shown in Figure A6, and the sensorgrams can be seen in Figure A7. Peptides 6 and 10 showed a higher signal for uPAR than for CD4, whereas the opposite trend was observed for peptide 7.

### 3.4. Peptide Persistence in Blood over Time

**Figure 4:**
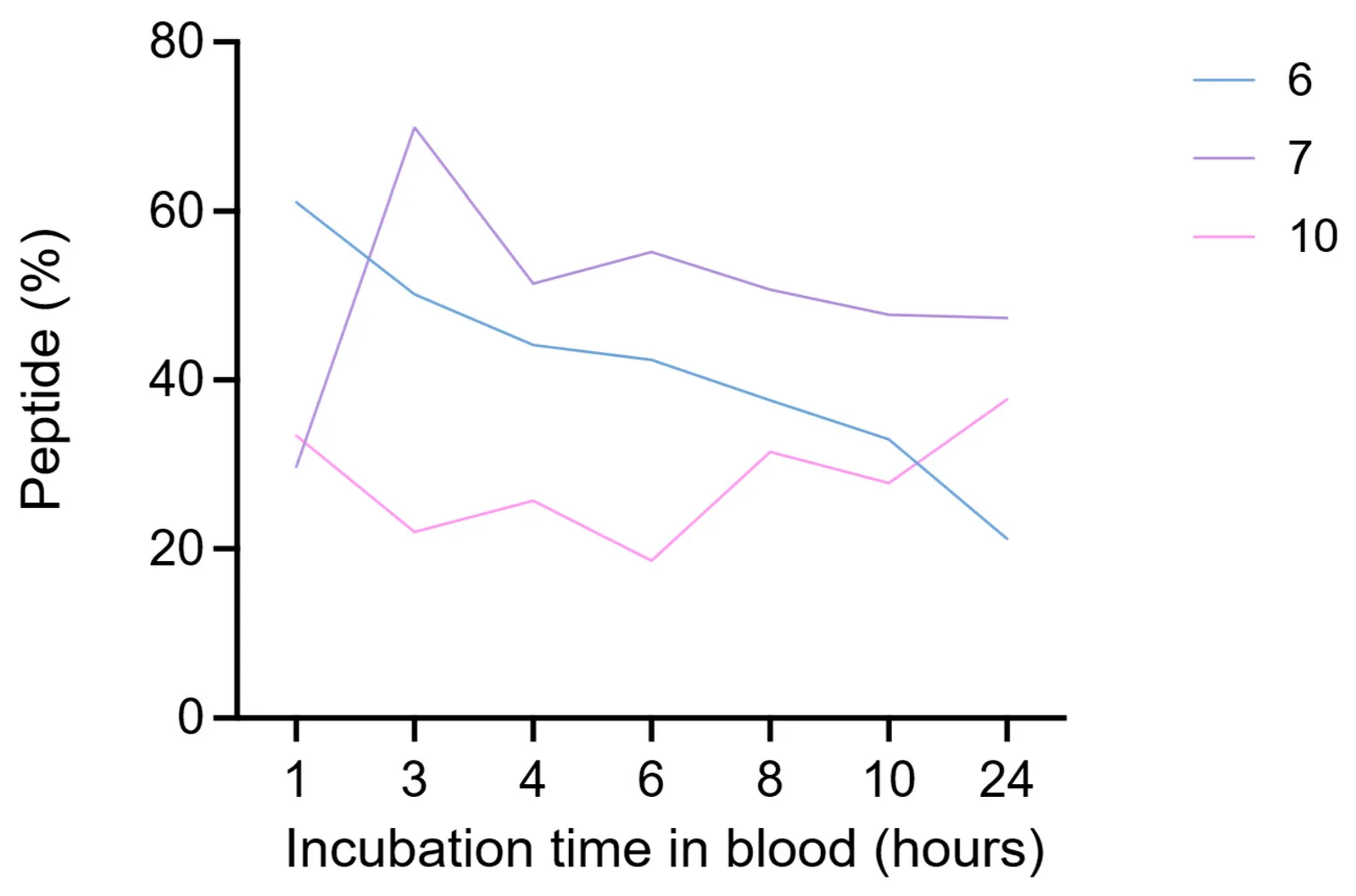
Peptide stability in blood. Peptides at the same concentration were incubated in the blood from a healthy individual for up to 24 h. The percentage of peptides was assessed by HPLC with a fluorescence detector.

The persistence of the three selected peptides in blood over time was investigated to assess whether they would potentially be suitable for downstream in vivo applications. All three peptides were incubated in whole blood of a healthy individual for up to 24 h and subsequently analyzed using HPLC with a fluorescence detector. The results are shown in Figure 4. Variations in the percentage of peptides can be observed throughout the experiment. After the first hour, the percentages of both peptides 7 and 10 are remarkably lower than those of peptide 6. Hereafter, peptide 6 exhibits a steady decrease throughout the experiment, whereas the pattern for peptides 7 and 10 exhibits more variation. The percentage of peptide 7 is the highest after 24 h.

### 3.5. Molecular Docking Suggests a Binding Pocket Shared with uPA

**Figure 5:**
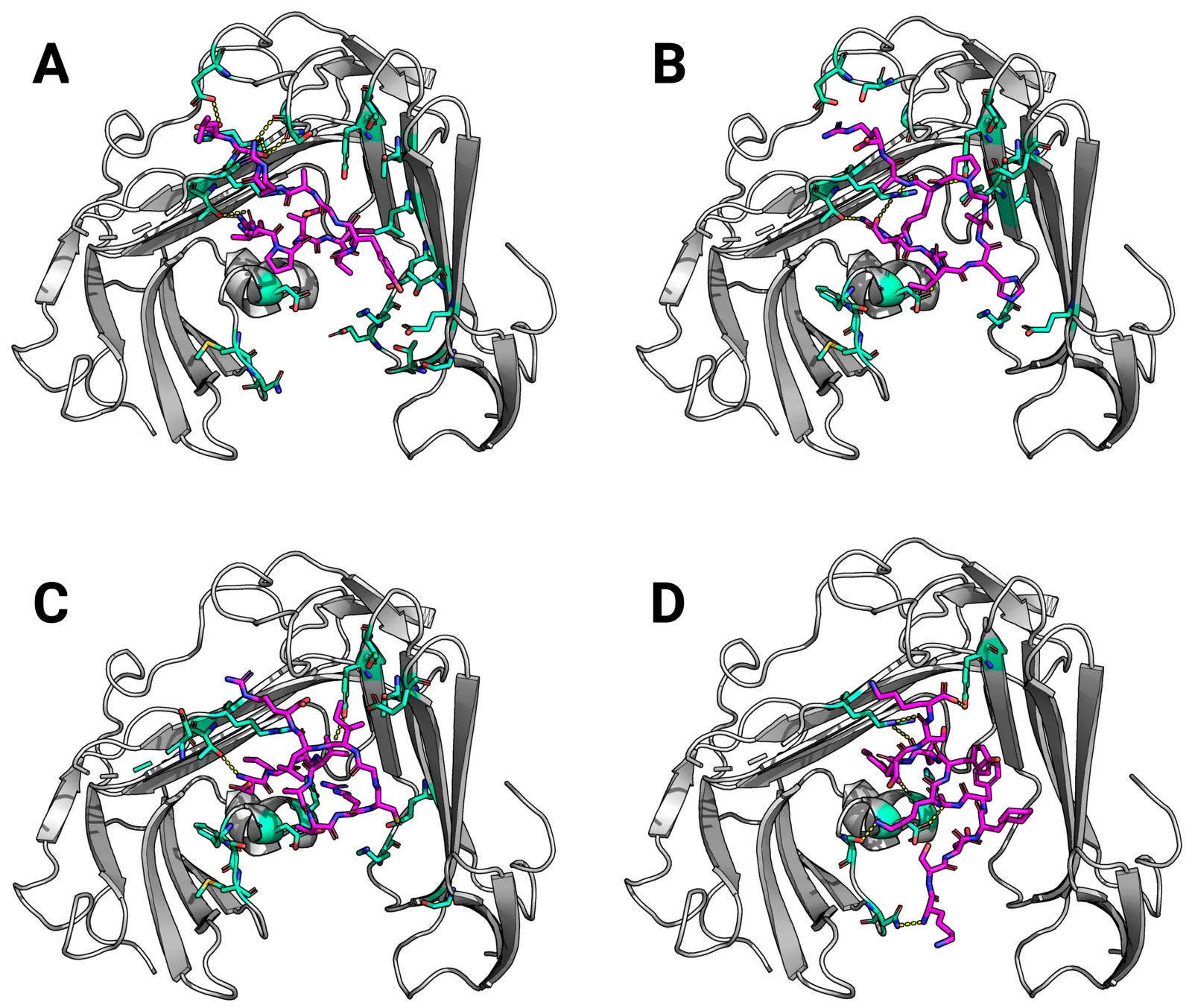
Molecular docking of the selected peptides (magenta) 6, 7, and 10 to uPAR (gray). The uPAR conformation selected for molecular docking was the crystal structure of the protein when bound to an AE105 derivative (AE147). The residues in cyan indicate uPAR residues within 3 Å of the peptide. The yellow dotted lines show 3 Å peptide-uPAR interactions identified using PyMOL based on the docked peptide poses generated by AutoDock Vina. (**A**) 6, (**B**) 7, (**C**) 10, (**D**) AE147. PDB: 1YWH.

Molecular docking of the selected peptides (6, 7, and 10) to uPAR was performed to explore where on the uPAR protein the identified peptides might bind, using a grid box covering the entire uPAR protein. The molecular docking results were compared to the known uPAR-binding 9-mer peptide, AE105 [28]. The uPAR structure used for docking was a crystal structure of the protein when bound to a derivative of AE105 (AE147), which exhibits a high resemblance to AE105 and contains the amino acids critical for binding to uPAR but has higher solubility [56]. All three peptides were predicted to dock within the same binding pocket of uPAR as AE147. The docking scores for the peptides presented in Figure 5 are −6.9 kcal/mol for peptide 6, −6.8 kcal/mol for peptide 7, and −5.8 kcal/mol for peptide 10.

## 4. Discussion

Phage display remains a powerful methodology for the identification of novel peptide ligands. However, challenges such as the enrichment of TUPs can complicate the translation of peptide leads into functional imaging probes or therapeutics [58,59,60,61]. In this study, we addressed these challenges using a combination of subtractive biopanning, serial amplification as biopanning selection controls, NGS, bioinformatic filtering, and multiple in vitro validation strategies to identify and characterize phage display-derived peptides and their binding to uPAR. This integration of target-directed selection and selection controls with multi-assay validation builds on and further extends beyond our prior methodological work on characterizing phage display libraries using NGS [42,44,45,46].

To reduce the risk of identifying TUPs, we implemented a subtractive biopanning strategy and included amplification controls of the unselected library. Together with bioinformatic filtering, this approach is essential to limit the number of propagation-related sequences that could otherwise falsely be included in downstream analyses [58,60]. By combining phage display with NGS and using a high sequencing depth, we were able to employ stringent filtering of the datasets. In a previous report, we demonstrated the importance of sequencing depth in the analysis of peptide phage display libraries [46]. As Sanger sequencing is traditionally used, it can be expected that many nonspecifically enriched peptide sequences may not be detected. By utilizing a high sequencing depth in this work, we could obtain a more comprehensive overview of propagation-related peptides. Despite the stringent bioinformatic filtering of the biopanning dataset, extensive validation of peptide hits remains essential following phage display selection.

A comprehensive validation strategy should confirm target specificity using both the target used for biopanning and cell-based assays, when relevant. In this study, peptide candidates were evaluated for binding to recombinant uPAR as well as uPAR expressed on the surface of live cells, ensuring that binding is also possible in a more physiologically relevant context. When sequencing of the phage clones is performed by Sanger sequencing, phage ELISA is a common method to assess binding properties. However, this approach is incompatible with NGS. Furthermore, validation using intact phage particles can yield false positives due to phage surface components mediating interactions with the target, rather than the displayed peptide. Therefore, it is important to validate peptide binding using synthesized peptides, ideally using several methods, to ensure that the interactions are peptide-specific.

Also, the use of recombinant uPAR protein immobilized on a plastic surface during biopanning introduces the caveat that the native conformation of the protein might not be preserved. Structural alteration and, depending on the expression host, lack of post-translational modifications can limit the translational potential of the identified peptides [62]. Hence, it is essential that the identified peptides also recognize the native form of the target of interest. To address this, we validated the selected peptides by different in vitro assays using cell lines differing in endogenous uPAR expression. While this approach offers the advantage of testing the peptides against the receptor in its native context, it introduces potential confounders arising from intercellular variability. The use of a CRISPR-Cas9-generated uPAR knockout cell line would provide a more controlled model for future validations.

Flow cytometry was performed to investigate the binding behavior of all dissolvable peptides to Jurkat and SUP-T1 cells with high and low uPAR expression, respectively. Here, it was found that peptide 10 showed a statistically significant difference in signal when comparing Jurkat and SUP-T1. We further explored the binding behavior of peptides 6, 7, and 10 by a plate-based assay, which, although potentially less sensitive than single-cell approaches like flow cytometry, allowed efficient screening of multiple peptide candidates. The results from the plate-based assay also showed binding of peptide 10; however, peptide 7 also exhibited statistically significant binding. The consistency between flow cytometry data and the plate-based assay results reinforced the robustness of the results.

Comparing the signal obtained with the anti-uPAR antibody using flow cytometry (Figure 2B and Figure A1) to that of the identified peptides (Figure 3A and Figure A4) reveals a larger separation in signal between Jurkat and SUP-T1 cells for the antibody compared to the peptides. This difference likely reflects the higher affinity of the monoclonal antibody compared to the phage display-derived peptides, which have not yet undergone any optimization, and may also reflect differences in the accessibility of the respective binding epitopes on uPAR. The calculation of the normalized binding was performed to circumvent potential confounding factors, such as a higher autofluorescence for one cell line, as well as inter-sample variance.

While post hoc comparisons identified a statistically significant difference in signal between Jurkat and SUP-T1 specifically for peptide 10 in both assays, the two-way ANOVA results differed between the two validation methods. For the flow cytometry data, the peptide × cell line interaction did not reach statistical significance (F(3,16) = 2.165, *p* = 0.1321), indicating that at the level of the model, the data do not provide strong statistical support for the claim that the magnitude of the difference between Jurkat and SUP-T1 varies across peptides. This underscores the importance of interpreting the post hoc result of peptide 10 with caution pending further validation with larger sample sizes. In contrast, the plate assay data showed statistically significant peptide × cell line interaction (F(3,16) = 3.511, *p* = 0.0397), providing stronger statistical support for peptide-dependent differences between the two cell lines in this assay. This discrepancy between assays may reflect differences in sensitivity, dynamic range, or signal-to-noise between single-cell flow cytometry and the plate-based fluorescence readout and reinforces the importance of evaluating peptide candidates using multiple complementary validation methods, as differences observed by one method may not be statistically robust across all approaches.

The effect of conjugating a peptide to a larger moiety, such as a fluorophore, should be carefully considered. Firstly, it may impede peptide binding to the target by steric hindrance, by partially masking the binding interface or restricting the peptide’s conformational flexibility. Moreover, due to the hydrophobic nature of FITC, it might render the peptide less soluble and more prone to aggregation. In this work, the peptides were synthesized with a linker expected to increase the solubility of the peptide conjugate. Additionally, the fluorophore itself must be suitable for the downstream assays. While FITC is widely used, it is sensitive to photobleaching, and cell autofluorescence can overlap with FITC emission [63,64], underscoring the importance of including proper controls, such as pure FITC, to identify potential non-specific binding.

SPR was employed to validate the binding behavior of the FITC-labeled peptides toward recombinant uPAR. Peptide 10 showed binding to uPAR and negligible binding to the reference protein, CD4, whereas peptide 7 showed the opposite pattern, with a higher signal for CD4 than for uPAR, despite showing a higher signal for Jurkat compared to SUP-T1 in both cell-based assays. This discrepancy may be explained by the differences between target presentation in the two assay formats, where SPR relies on the recombinant uPAR immobilized via random amine coupling to a sensor chip, which can affect the receptor orientation and reduce the availability of the native binding epitope, whereas in cell-based assays the compounds are presented to uPAR in its native, membrane-anchored form [65]. While SPR is considered a sensitive platform [66], it necessitates that the receptors are in their native conformation and have a uniform orientation with a binding site available for the flow of analytes, which is not guaranteed when using random amine coupling. It could be hypothesized that some proportion of the immobilized uPAR protein is in a conformation that is not recognized by peptide 7, which could account for the reduced SPR signal. This underscores that a discordant SPR result does not necessarily rule out target engagement, and reinforces the value of evaluating peptide binding across assay formats employing different modes of target presentation.

Taken together, the cell-based assays and SPR provided complementary but not fully concordant evidence of the binding properties of the peptide candidates, a pattern likely reflecting fundamental differences between the assay formats. This underscores that no single assay format should be treated as a definitive measure of binding. The multi-assay validation strategy applied in this study, combined with stringent NGS-based bioinformatic filtering at the selection stage, offers a more robust and reproducible framework for candidate prioritization than relying on a single validation method.

The persistence of the FITC-related signal for the peptides in blood was evaluated to gain preliminary insight into their potential suitability for future in vivo applications, such as imaging or drug delivery. As HPLC with fluorescence detection cannot distinguish intact peptide from degraded products still containing FITC, these results should be interpreted as an indication of the signal persistence rather than a confirmation of peptide integrity. Peptide 7 displayed a peak at the beginning of the experiment but remained relatively stable from 4 h of incubation up until 24 h, while peptide 10 exhibited more variable signal over time. These observations suggest that the FITC signal for both peptides 7 and 10 persisted over the 24 h. However, additional confirmation, such as LC-MS, is necessary to establish whether the peptide is still in its intact full-length form before drawing conclusions regarding its suitability for in vivo applications.

Molecular docking indicated that the peptides converge on a binding region shared with the known uPAR ligand, AE105. Notably, the docking grid (58 Å × 50 Å × 56 Å) encompassed the entire uPAR domain, allowing each peptide to dock to any accessible site on the receptor surface without the bias of a predefined region. The consistent convergence of all three peptides on the same pocket occupied by AE105/AE147, despite this unconstrained search space, therefore provides a more meaningful indication of preferential engagement with this region of uPAR. However, the docking results should be interpreted as a structural hypothesis suggesting a possible binding mode, which would require experimental validation, e.g., through competitive binding assays with AE105. Notably, AE105 was originally identified through a phage display selection against uPAR, but subsequently underwent several rounds of affinity maturation by combinatorial chemistry to improve its binding affinity and to identify the functional epitope [28]. In contrast, the peptides identified in this study are primary phage display hits that have not yet undergone comparable optimization, suggesting that the peptides identified in this study could potentially be further improved through such affinity maturation strategies.

Overall, the stringent bioinformatic analyses and in vitro validation strategy in our study identified peptide 10 as the leading peptide candidate, showing the most consistent binding behavior across different validation methods, including flow cytometry, plate-based assay, and SPR. Peptide 7 also showed binding in the plate-based assay and had a relatively high FITC signal in blood, whereas peptide 6 showed less consistent evidence of binding across the assays. The differences between the validation methods illustrate that enrichment during phage display alone is insufficient to establish target binding, and that candidate peptides should be evaluated using complementary approaches. Based on the results, peptide 6 provides less evidence for uPAR binding, while peptide 7 may warrant additional evaluation. From the results obtained in this study, peptide 10 represents the strongest candidate for further investigations.

## 5. Study Limitations

The binding of the identified peptides was evaluated primarily by comparing signal between Jurkat and SUP-T1 cells, which differ in endogenous uPAR expression. While this difference is consistent with uPAR-dependent binding, the two cell lines may also differ in numerous other surface receptors, adhesion molecules, and membrane properties that can influence peptide binding or fluorescence readout independently of uPAR expression. As such, the present data do not unequivocally establish uPAR-specific binding. A more controlled system, such as uPAR knockout cells, uPAR overexpressing cells, or competition assays, would be required to more definitively establish uPAR dependence and is recommended for future validation of these candidates.

The peptides used in the cell-based binding assays were dissolved in PBS containing up to 10% (*v*/*v*) MeCN to improve the peptide solubility. As organic solvents at this concentration may cause potential solvent-related effects on cell behavior, for example, changes in membrane properties, the potential contribution of solvent-related effects to the observed signal cannot be fully excluded. The binding results should therefore be interpreted in the light of these experimental conditions, and confirmation using lower MeCN concentrations or alternative solubilization strategies would strengthen the conclusions.

The SPR analysis performed in this study has several limitations that should be considered when interpreting the reported binding results. Peptide 10, the primary candidate, was tested only once on the uPAR surface due to precipitation, limiting the reliability of this specific measurement. The experimental setup did not include a scrambled peptide. uPAR and CD4 were immobilized at different levels (900–1100 RU and 600 RU, respectively), which complicates the comparison of response amplitudes between the two surfaces. The SPR results presented are preliminary, and a more complete concentration series, independent experimental repeats, and quantitative kinetic analysis would be required.

The blood stability assay showed a pattern of inconsistent decrease in FITC-associated signal for peptides 7 and 10, rather than the expected steady decline due to proteolytic degradation. This could suggest possible technical artifacts (i.e., precipitation, variability in sample processing). Furthermore, fluorescence detection cannot distinguish intact peptides from fragments containing FITC. To confirm the extent of peptide degradation over time, LC-MS should be included in future studies.

The molecular docking analysis performed in this study should be interpreted as a hypothesis rather than as a confirmation. The docking grid encompassed the entire uPAR, allowing each peptide to dock to any accessible site on the receptor surface without bias toward a predefined binding pocket. The docking was based on a single crystal structure of uPAR (PDB: 1YWH) and computationally predicted peptide conformations, which do not accurately account for receptor or peptide flexibility, solvent effects, or the dynamic nature of protein-peptide interactions. The predicted binding poses represent a structural hypothesis regarding a possible mode of peptide-uPAR engagement, which would require experimental validation, i.e., through AE105 competitive binding assays.

## 6. Conclusions

In this study, phage display in combination with NGS-based selection and bioinformatic filtering was exploited to identify candidate peptides targeting uPAR. Peptide candidates were identified and evaluated using complementary in vitro assays, including flow cytometry, quantitative plate assay, SPR, and molecular docking. Of the three peptides, some showed evidence of binding to uPAR-expressing cells and recombinant uPAR, with peptide 10 showing the most consistent behavior across validation methods and representing the strongest candidate for further investigation. The present data do not establish unequivocal uPAR-specific binding, and additional experiments, such as competition assays or the use of uPAR knockout or overexpressing models, would be required to confirm this. A blood stability assay indicated that the FITC-related signal for peptides 7 and 10 persisted over a 24 h incubation period. However, before drawing conclusions regarding suitability for in vivo applications, additional studies are necessary to confirm the integrity of the peptide. Overall, this study demonstrates a workflow combining optimized phage display selection with NGS and multi-assay validation for the identification of candidate peptides, and highlights peptide 10 as the most promising candidate warranting further optimization, including affinity maturation and additional target-specificity studies, toward potential applications in cancer imaging and therapy.

## Figures and Tables

**Table 1:** Overview of the results from quality filtering of the biopanning samples. The number of cleaned and removed reads and the percentages are shown. Furthermore, the number of reads being removed due to the presence of ‘X’ or ‘*’ in the reads or the absence of the GGGS-linker is shown.

	Naïve Library	uPA Elution Round 1	Acid Elution Round 1	uPA Elution 2	Acid Elution Round 2
Total reads	5.3 × 10^6^	6.2 × 10^6^	1.3 × 10^7^	5.5 × 10^6^	3.5 × 10^6^
Cleaned reads	1.3 × 10^6^	1.2 × 10^6^	4.2 × 10^6^	1.5 × 10^6^	1.1 × 10^6^
Cleaned reads (%)	24	19	32	28	32
Removed reads	4.0 × 10^6^	5.0 × 10^6^	8.9 × 10^6^	4.0 × 10^6^	2.4 × 10^6^
‘X’ and ‘*’	7.1 × 10^4^	5.5 × 10^4^	5.1 × 10^4^	6.4 × 10^4^	1.7 × 10^4^
No GGGS	8.1 × 10^4^	2.3 × 10^5^	2.7 × 10^5^	2.9 × 10^4^	2.1 × 10^4^

**Table 2:** Overview of peptides selected based on the first round of biopanning. The type of elution employed, the enrichment factor (EF), and the relative peptide frequencies in both the biopanning eluate and in the unselected library are shown.

Peptide	Elution Type	EF	Relative Frequency in Biopanning Eluate	Relative Frequency in Unselected Library
1	uPA elution and acid elution	14,731.6	0.01325	8.99 × 10^−7^
2	uPA elution and acid elution	12,981.1	0.01167	8.99 × 10^−7^
3	uPA elution and acid elution	8093.8	0.00728	8.99 × 10^−7^
4	uPA elution	1079.7	0.00097	8.99 × 10^−7^
5	uPA elution	895.7	0.000805	8.99 × 10^−7^
6	uPA elution	737.7	0.00066	8.99 × 10^−7^
7	uPA elution	100.3	9.02 × 10^−5^	8.99 × 10^−7^
8	uPA elution	97.6	8.77 × 10^−5^	8.99 × 10^−7^
9	uPA elution	180.3	0.00016	8.99 × 10^−7^
10	uPA elution	303.8	0.00027	8.99 × 10^−7^
11	uPA elution	638.3	0.000574	8.99 × 10^−7^

## Data Availability

Dataset available on request from the authors.

## Abbreviations

The following abbreviations are used in this manuscript:

BDB	Biopanning Data Bank
BSA	Bovine serum albumin
cDNA	Complementary DNA
CRISPR	Clustered Regularly Interspaced Short Palindromic Repeats
DNA	Deoxyribonucleic acid
ECM	Extracellular matrix
*E. coli*	*Escherichia coli*
EDTA	Ethylenediaminetetraacetic acid
EF	Enrichment factor
ELISA	Enzyme-linked immunosorbent assay
FACS	Fluorescence-activated cell sorting
FDG	Fluorodeoxyglucose
FITC	Fluorescein isothiocyanate
FI	Fluorescence intensity
GGGS	Glycine-glycine-glycine-serine linker
HPLC	High-performance liquid chromatography
IPTG	Isopropyl β-D-1-thiogalactopyranoside
LB	Luria–Bertani medium
MeCN	Acetonitrile
MFI	Median fluorescence intensity
NGS	Next-generation sequencing
PBS	Phosphate-buffered saline
PCR	Polymerase chain reaction
PDB	Protein Data Bank
PEG	Polyethylene glycol
PE	Phycoerythrin
PET	Positron emission tomography
pfu	Plaque-forming unit
PLAUR	Urokinase plasminogen activator gene
Pr-TUP	Propagation-related target-unrelated peptide
qPCR	Quantitative polymerase chain reaction
RNA	Ribonucleic acid
SAROTUP	Scanner and Reporter of Target-Unrelated Peptides
SD	Standard deviation
SEM	Standard error of the mean
SPR	Surface plasmon resonance
Sr-TUP	Selection-related Target-Unrelated Peptides
ssDNA	Single-stranded DNA
TBS	Tris-buffered saline
TBST	Tris-buffered saline with Tween-20
TCA	Trichloroacetic acid
TUP	Target-Unrelated Peptide
uPA	Urokinase plasminogen activator
uPAR	Urokinase plasminogen activator receptor
Xgal	5-Bromo-4-chloro-3-indolyl-β-D-galactopyranoside

## Appendix A

**Table A1:** Overview of predictions of peptides. Predictions of peptide solubility were made, and MimoBlast was used to determine whether the peptides had previously been identified in other phage display experiments. Neither of the peptides had been found in previous biopanning experiments, according to the Biopanning Data Bank.

Peptide	Predicted Solubility	MimoBlast Results
1	Poor	
2	Good	Target: anti-neuwiedase polyclonal antibodies
3	Good	
4	Good	
5	Good	Target: Anti-MCPS monoclonal antibody 1E4
6	Poor	Target: serine-aspartate repeat-containing protein G
7	Good	
8	Poor	
9	Poor	Target: semiconductor crystalline surface CdSe(100)
10	Good	
11	Good	
